# Health and wealth in children and adolescents with chronic kidney disease (K-CAD study)

**DOI:** 10.1186/1471-2458-14-307

**Published:** 2014-04-04

**Authors:** Germaine Wong, Meredith Medway, Madeleine Didsbury, Allison Tong, Robin Turner, Fiona Mackie, Steven McTaggart, Amanda Walker, Sarah White, Kirsten Howard, Siah Kim, Jonathan C Craig

**Affiliations:** 1 Centre for Kidney Research, Kids Research Institute, The Children’s Hospital at Westmead, Sydney, Australia; 2 Sydney School of Public Health, The University of Sydney, Sydney, Australia; 3 Centre for Transplant and Renal Research, Westmead Hospital, Sydney, Australia; 4 Department of Biostatistics, University of New South Wales, Sydney, Australia; 5 Department of Renal Medicine, Sydney Children’s Hospital at Randwick, Sydney, Australia; 6 Department of Renal Medicine, The Royal Children’s Hospital, Brisbane, Australia; 7 Department of Renal Medicine, The Royal Children’s Hospital, Melbourne, Australia; 8 Sydney Medical School, The University of Sydney, Sydney, Australia

**Keywords:** Socioeconomic status, Chronic kidney disease, Quality of life, Mortality, Cohort studies, Qualitative

## Abstract

**Background:**

The impact of reduced kidney function in children is substantial. End-stage kidney disease (ESKD), the most severe form of chronic kidney disease (CKD), is a devastating illness associated with substantially increased mortality, impaired growth and psychosocial maladjustment in children. Understanding how to address the complex causes of mortality and morbidity in children with CKD requires explicit information about the risk factors that lead to adverse outcomes. In addition to biological influences, the socioeconomic circumstances of caregivers may play a significant role in the health and well-being of children with CKD.

**Methods/Design:**

A prospective cohort study (n = 380 children and n = 380 caregivers) will be conducted to determine the prevalence of economic hardship among caregivers of children with CKD. All participants will be followed biennially over a period of 5 years to determine the association between the changing socioeconomic status of the caregivers and the health and overall well-being of school-aged children with CKD. Face to face, semi-structured interviews with the caregivers (n = 45) will also be conducted to understand their perspectives on the economic, financial and psychosocial impact of CKD and how this affects the health outcomes of their child with CKD. The primary outcomes of the study are the effects of the socioeconomic status of the caregivers and self-reported health status of the children. Secondary outcomes included the prevalence of economic hardship and the distribution of wealth among the caregivers of children with CKD.

**Discussion:**

Findings from this study presents not only a snapshot of the current economic and social situation of the caregivers of children and adolescents with CKD but will also provide definitive evidence of determining whether a link between socioeconomic status of caregivers and outcomes of children with CKD exists.

## Background

Children and adolescents with chronic kidney disease (CKD) are at risk of dying prematurely, predominantly from cardiovascular disease. The mortality rate of children with CKD requiring renal replacement therapy in the form of dialysis or kidney transplantation is at least 30-fold higher than their age-matched peers, and health outcomes are not improving [1]. Children undergoing dialysis suffer significant disruption to their daily routine and quality of life. These children are attached to a machine that filters toxins from their blood at least 4–5 hours per day. They may have significant fluid and dietary restrictions imposed in order to manage electrolyte disturbances and fluid overload. Although transplantation is the preferred form of renal replacement therapy, children with kidney transplants are reliant on long-term maintenance immunosuppression, which is associated with adverse long-term effects such as increased infection and risk of cancer. Apart from physical health, CKD also negatively impacts on the overall psychosocial, cognitive and emotional well-being of the child [2-5].

CKD is usually an irreversible, progressive disease, and if left untreated, can lead and in most cases will progress to end-stage kidney disease requiring renal replacement therapy such as dialysis and transplantation. Several known and potentially modifiable risk factors are responsible for disease progression in children and adolescents with CKD, such as blood pressure and proteinuria [6]. Apart from these known modifiable risk factors for renal progression, socioeconomic status may also play a role for CKD progression in children.

### Socioeconomic status and chronic disease in children and adolescents

Despite the concerted effort by government and policy-makers to close the gap between the rich and the poor, the social and health inequality between the most affluent and the under-privileged remains. Recent research shows that children and adolescents from economically disadvantaged backgrounds have a higher prevalence of asthma and associated co-morbidities such as antibiotic resistant upper respiratory infections [7]. The rates of bronchitis were at least 182% higher among children from the lowest quintile of equivalence income compared to the highest quintile. The current report from the Australian Institute of Health and Welfare indicates that children from lower socioeconomic backgrounds are more likely to take days off from school due to sickness compared to those with parents from an affluent background. In addition, adolescents from lower socioeconomic backgrounds are less likely to engage in health preventive strategies such as sun protection and regular dental consultations, and are more likely to be involved in adverse health-related behaviours such as smoking and excessive alcohol drinking [8]. A better understanding and accurate assessment of the causal pathway between the socioeconomic determinants and health outcomes in children is critical for the development of appropriate intervention that effectively reduces the social and economically disadvantaged health disparities in children with chronic disease.

### Hypotheses and overall aim of the study

The range of socioeconomic determinants that influences the quality of life of these children is broad, and includes household income, parental occupation, mother’s education, level of health insurance, and marital status. Despite the current evidence suggesting a direct link between socioeconomic status and the severity of CKD in adult CKD patients [9], a similar relationship between socioeconomic status of the caregivers and the health outcomes of children and adolescents with CKD has not been established. Addressing the social determinants of health is a primary approach to achieving health equity. Understanding and expanding the knowledge base of how social and economic factors may have on the health outcomes in children with CKD is critical to influence program and policy activities, build partnership between government and healthcare workers, and to develop tailored interventions that target resource distribution to eliminate health disparities.

The aims of our study are:

1. To estimate the prevalence of economic hardship among caregivers of children with CKD

2. To determine the relationship between socioeconomic status of the caregivers and overall health outcomes and well-being of children with CKD

3. To describe caregivers perspectives on the economic and financial impact of caring for school-aged children with CKD

## Methods/Design

The KCAD study is a prospective cohort study that assesses the changing socioeconomic status of the caregivers and the health and well-being of children with CKD. This research will take place in four different sites (Sydney Children’s Hospital at Randwick, The Children’s Hospital at Westmead, Royal Children’s Hospital in Brisbane, The Royal Children’s Hospital in Melbourne) in Australia. The design, conduct and reporting of the study is in accordance with the Strengthening the Reporting of Observational Studies in Epidemiology (STROBE) initiatives for cohort studies [10].

### Eligibility and study population

All school aged children (ages 6 – 18) with CKD stages I-V, on dialysis and with kidney transplants (and their primary caregivers) will be invited to participate in the study. Caregivers and/or children who may be medically unfit, unable to provide written informed consent and children/adolescents who are not receiving any formal education will be excluded.

### Data collection

The following will be collected in two separate self-reported questionnaires by the primary caregivers and the child with CKD at baseline and biennially up to a follow-up period of four years.

1. *Caregivers:* age, gender, ethnicity, other medical co-morbidities and marital status of the primary caregivers.

2. *Children and adolescents with CKD*: age, gender, ethnicity, medications, immunosuppression use, other medical co-morbidities such as hypertension and cardiovascular disease, duration on dialysis.

In addition to these factors, the socioeconomic factors of the caregivers will be measured by: post-codes, real annual disposable household income, equivalised total income (measure of material living standards, obtained by adjusting household disposable income for the household’s ‘needs’). Other factors that may affect the financial and economic status of the household include: age and gender of household members, health and disability of household members, region of residence and home-ownership status, estimated total assets and liabilities, household composition, household size, health insurance coverage, change in household income since the diagnosis of CKD of the child, and the education status of the caregivers. The index of relative socioeconomic disadvantage scores (IRSD) will also be generated for the families based upon the corresponding post codes of their residences. The IRSD is part of the SEIFA scores that assess the different aspects of socio-economic conditions by geographic area. In addition to objective questions about income and socioeconomic status, a validated question about subjective income poverty will also be asked: "Given your current needs and financial responsibilities, would you say that you and your family are (1) Prosperous; (2) Very comfortable; (3) Reasonably comfortable; (4) Just getting along; (5) Poor; or (6) Very poor?".

### Outcome measures

The primary outcome of the study is the self-rated health status reported by school aged children with CKD. Self-reported health status is a validated, subjective tool that had been used to measure the overall general health and to predict mortality in adults and children with chronic illnesses [11]. The perceived health state by the patients has been shown to be a reliable surrogate marker of the underlying health state and disease burden experienced by the patients. Participants will be asked the following question: "Would you say your health in general as excellent, very good, good, fair or poor?"

The secondary outcomes include: 1) the prevalence of economic hardship among caregivers of school-aged children and adolescents with CKD. Economic hardship is measured using a series of questions about financial stress (e.g. failure to pay basic living and medical expenses) and the use of dissaving actions (e.g. reducing home loan repayments, increasing balance owed on credit cards, selling assets, taking out a personal loan, etc.) in the previous 12 months, 2) the distribution of wealth among the caregivers of school-aged children and adolescents with CKD, 3) quality of life (QoL) assessment: the self-conducted Health Utility index (HUI) will be used to assess the utility-based QoL of the child, 4) physical health – i) progression of CKD: measure as changes in eGFR (estimated using the modified Schwartz formula) using the over the follow-up period (for those not on dialysis), ii) acute rejection and graft failure rates, iii) patient and renal graft survivals (for kidney transplant recipients), iv) child’s physical growth – measuring height and weight 5) educational outcomes: National Assessment Program – Literacy and Numeracy (Naplan) scores and self-reported school grades (categories as the five common grade scales of: elementary, basic, satisfactory, substantial and excellent), and the intelligent quotient (IQ) score (using the Weschler Intelligence Scale for Children). The Naplan test is a compulsory assessment of all students in years 3, 5, 7 and 9 studying within Australia that evaluates a combination of skills involving four major domains: numeracy, writing, reading and language conventions.

To ensure adequate follow-up of all outcomes such as graft and patient survival, we will compare our records to the Australian and New Zealand Dialysis and Transplant (ANZDATA) registry for all our patients who are on renal replacement therapies (dialysis and transplant) through data linkage at 2 and 4 years after enrolment to the study. Informed consent will be obtained from the caregivers and our school-aged children for the data-linkage process. The ANZDATA registry is a comprehensive registry that collects and reports annually on the incidence, prevalence and major outcomes such as graft, patient and cancer survival of dialysis and transplant treatment for patients with end-stage kidney disease within Australia and New Zealand.

### Sample size and statistical analyses

Prevalence epidemiological studies have suggested the overall population prevalence of economic hardship in the Australian population is 10.7% compared to 30% among those with chronic illness [8,11]. Assuming a confidence level of 95%, the margin of error at 0.05, the estimated prevalence of economic hardship of 30% among caregivers of school aged children with CKD, a total of 323 school-aged children and their caregivers will be needed to estimate the prevalence of economic hardship among the caregivers of school aged children with CKD. Data from our pilot study suggested that 30% of the school aged children with CKD rated their current health as fair or poor among those with caregivers of the lowest income quartile, compared to 13% for those whose primary caregivers were at the highest income quartile.

To investigate the association between the socioeconomic status of the caregivers and the self-rated health of school-aged children and adolescents with CKD, a sample size of 378 children will achieve 80% power to detect an effect size of 0.17 using three degrees of freedom Chi-square test with a significance level (alpha) of 0.05. Therefore a conservative estimate of 380 children and their caregivers will be needed for our proposed program of research. If we expect a participation rate of 50%, a target population of 756 school aged children and their caregivers will be required. The age-specific prevalence of economic hardship among the caregivers of school-aged children and adolescents will be measured and compared with the Australian general population using data from the Australian Bureau of Statistics. Hardship will be constructed as a dichotomous variable where a reported inability to make any of the payments posed or the use of a dissaving action will be classed as a case of hardship. Univariate and multivariate regression modeling will be conducted to assess the relationship between the socioeconomic status of the caregivers at baseline and the health and well-being of the school-aged children and adolescents with CKD. For continuous health outcomes such as utility-based quality of life scores, we will use the ordinary least squares. For categorical outcomes such as death or self-rated health, we will use logistic regressions.

Similar models will also be constructed to assess the changing socioeconomic status of the caregivers over time and the health and well-being of the school-aged children and adolescents with CKD. Four different socioeconomic trajectories of the caregivers will be created: persistent low socioeconomic status over time, persistent high socioeconomic status, downward mobile from high to middle or lower socioeconomic status and upward mobile from lower or middle to higher socioeconomic status over time. Predictions derived from each of the four mobile courses will be tested in the logistic multiple regression framework. In each regression model, we will estimate the effects of changing socioeconomic status of the caregivers on the child’s health while controlling for other risk factors such as age, gender and ethnicity.

### Qualitative study

A qualitative sub-study will be conducted to determine the economic, financial and psychosocial impact on parents and caregivers caring for a child with CKD, and to identify the potential reasons for associations between social determinants and health outcomes. We will conduct face-to-face, semi-structured interviews with a minimum of 45 participants. Parents or caregivers of children with CKD (early chronic kidney disease, dialysis, transplantation) will be recruited from the four centres enrolled in this study. A purposive sampling strategy will be applied where participants will be identified by recruiting nephrologists to capture a range of socio-economic status, geographical location, ethnicity; and their child’s CKD stage, gender and age. Participants will be recruited until theoretical saturation (i.e. when little or no new concepts are being identified in subsequent interviews) has been achieved within each stage of CKD (minimum n = 15 to 20 per CKD stage). The interview guide will focus on: 1) how CKD has affected their household financial and economic status, 2) the financial impact of CKD on quality of life, and 3) the financial impact on providing care and accessing services, 4) financial impact of the health and well-being of the child and family, and 5) potential services that may help to improve their current financial situation. Also, as 20% of caregivers have been a living kidney donor for their child, the interview will also examine the financial effects of donation on caregivers as well as how this may affect the care of their child. All interviews will be recorded and transcribed verbatim. Transcripts will be analysed using the principles of grounded theory and thematic analysis. The quality of life of caregivers using the self-administered Health Utility index (HUI) will also be assessed at the time of the interview [12]. The findings will provide comprehensive and detailed insight into parental perspectives on their own financial situation and its meaning and impact in the context of caring for the child with CKD and the family. This can inform multidisciplinary health services to alleviate the financial impact and burden so parents are better empowered to provide care for their family, and particularly for their child with CKD.

### Ethical considerations

Participants will be informed (in writing and verbally) the aims, methods, and the measurements performed during this study. They will also be informed about the ethical issues such as confidentiality, and their rights of withdrawal from the study at any time. All participants will receive written information about the study (including the recruitment and follow-up process). If they agree to participate, an informed written consent will be signed by the participants. In all circumstances, the research will adhere to the criteria of the World Medical Association Declaration of Helsinki. This study has been approved by the Human Research Ethics Committee (HREC) of the Sydney Children’s Hospital Network. (*HREC approval no: 12SCHN159*).

### Discussion

CKD is a silent disease. Children with CKD do not experience symptoms until they have severe renal dysfunction. Once the symptoms develop, it is a devastated occurrence for the family and the child. These children suffer from a multitude of physical, mental and psychological complications. Apart from reduced life expectancy compared to those without kidney disease, they are confronted with a diverse range of adverse health and social outcomes including, stunted growth, failure to thrive, poor self-esteem, relationship, behavioural and learning problems, delayed and motor skills development.

Poverty remains a reality even in rich countries like Australia. Over the past decades, the income gap between families has widened substantially, and is even more pronounced in families with children who have chronic disease. The negative impact of poverty and all levels of health and educational outcomes have been widely demonstrated in children with other chronic illnesses such as epilepsy and asthma. The critical question remains – is social and economic disadvantage associated with worse health outcomes in children with CKD and are these consequences reversible or preventable by intervening to ameliorate and compensate the impact of being disadvantaged? Our proposed program of work provides not only a snapshot of the current economic and social situation of the caregivers of children with CKD, but will also examine the contextual differences between income and wealth distribution of their caregivers and outcomes in CKD. Understanding and recognising the needs of socially and financially disadvantaged families with CKD is vital to ensure an equitable and transparent service and budget planning for the underprivileged children with chronic illness and special health needs. It is envisaged that findings from our proposed program of research will provide a comprehensive analysis to allow development of a coordinated short and long-term care package and infrastructure to provide the much needed benefits and assistance for families of children and adolescents with CKD.

## Competing interests

The authors declare they have no competing interests.

## Authors’ contributions

Authors GW had the main role in manuscript preparation. Authors GW, JC, AT, SK, MW, SM and FM were responsible for research protocol design, while author MM and MD have the main role in data collection. Authors GW, KH and RT are responsible for statistical analyses and sample size calculations. All authors edited, read and approved the manuscript.

## Pre-publication history

The pre-publication history for this paper can be accessed here:

http://www.biomedcentral.com/1471-2458/14/307/prepub
